# Genetic controls of short- and long-term stomatal CO_2_ responses in *Arabidopsis thaliana*

**DOI:** 10.1093/aob/mcaa065

**Published:** 2020-04-16

**Authors:** Karin S L Johansson, Mohamed El-Soda, Ellen Pagel, Rhonda C Meyer, Kadri Tõldsepp, Anders K Nilsson, Mikael Brosché, Hannes Kollist, Johan Uddling, Mats X Andersson

**Affiliations:** 1 Department of Biological and Environmental Sciences, University of Gothenburg, Gothenburg, Sweden; 2 Institute of Technology, University of Tartu, Tartu, Estonia; 3 Department of Genetics, Faculty of Agriculture, Cairo University, Cairo, Egypt; 4 Department of Molecular Genetics, Leibniz Institute of Plant Genetics and Crop Plant Research (IPK) Gatersleben, Seeland, Germany; 5 Organismal and Evolutionary Biology Research Programme, Faculty of Biological and Environmental Sciences, University of Helsinki, Helsinki, Finland

**Keywords:** *Arabidopsis thaliana*, C24, CO_2_ response, stomata, stomatal conductance, *g*_s_, stomatal regulation, QTL mapping, RIL, NIL, water-use efficiency, water economy

## Abstract

**Background and Aims:**

The stomatal conductance (*g*_s_) of most plant species decreases in response to elevated atmospheric CO_2_ concentration. This response could have a significant impact on plant water use in a future climate. However, the regulation of the CO_2_-induced stomatal closure response is not fully understood. Moreover, the potential genetic links between short-term (within minutes to hours) and long-term (within weeks to months) responses of *g*_s_ to increased atmospheric CO_2_ have not been explored.

**Methods:**

We used *Arabidopsis thaliana* recombinant inbred lines originating from accessions Col-0 (strong CO_2_ response) and C24 (weak CO_2_ response) to study short- and long-term controls of *g*_s_. Quantitative trait locus (QTL) mapping was used to identify loci controlling short- and long-term *g*_s_ responses to elevated CO_2_, as well as other stomata-related traits.

**Key Results:**

Short- and long-term stomatal responses to elevated CO_2_ were significantly correlated. Both short- and long-term responses were associated with a QTL at the end of chromosome 2. The location of this QTL was confirmed using near-isogenic lines and it was fine-mapped to a 410-kb region. The QTL did not correspond to any known gene involved in stomatal closure and had no effect on the responsiveness to abscisic acid. Additionally, we identified numerous other loci associated with stomatal regulation.

**Conclusions:**

We identified and confirmed the effect of a strong QTL corresponding to a yet unknown regulator of stomatal closure in response to elevated CO_2_ concentration. The correlation between short- and long-term stomatal CO_2_ responses and the genetic link between these traits highlight the importance of understanding guard cell CO_2_ signalling to predict and manipulate plant water use in a world with increasing atmospheric CO_2_ concentration. This study demonstrates the power of using natural variation to unravel the genetic regulation of complex traits.

## INTRODUCTION

Stomata are microscopic pores in the epidermis, surrounded by two guard cells that regulate their aperture by changes in turgor pressure. Almost all gas exchange between plants and the atmosphere occurs through the stomata, hence the stomatal aperture is regulated to balance the trade-off between CO_2_ uptake for photosynthesis and transpirational water loss. Elevated CO_2_ concentration induces partial closure of stomata in most plant species (Morison, 1998; Ruszala *et al.*, 2011; Franks and Britton-Harper, 2016). This reduces transpirational water loss and improves leaf-level water economy. With a projected doubling of the atmospheric CO_2_ concentration within the next 100 years (IPCC, 2013), the stomatal CO_2_ response could have a significant impact on global plant water use under future climatic conditions. However, the magnitude of the stomatal CO_2_ response and hence the potential for water conservation under elevated CO_2_ exhibit a large variation among and within species (Morison, 1998; Takahashi *et al.*, 2015; Hõrak *et al.*, 2017). Significant variation in the stomatal CO_2_ response among different accessions of the model plant *Arabidopsis thaliana* (Takahashi *et al.*, 2015) provides an excellent opportunity to explore its genetic basis, as indicated by the recent discovery of a novel CO_2_ signalling component using natural *A. thaliana* accessions (Jakobson *et al.*, 2016). Knowledge about the genetic regulation of stomatal conductance (*g*_s_) in response to elevated CO_2_ could facilitate the improvement of crop water-use efficiency in a future climate.

The pathway for stomatal closure in response to elevated CO_2_ consists of one CO_2_-specific branch that converges downstream with the pathway for abscisic acid (ABA)-induced stomatal closure (Webb and Hetherington, 1997; Engineer *et al.*, 2016). The CO_2_ response is initiated by the conversion of CO_2_ to bicarbonate by the carbonic anhydrases CA1 and CA4 in guard cells (Hu *et al.*, 2010), resulting in the activation of the mitogen-activated protein kinases MPK4 and MPK12 by a yet undescribed mechanism (Marten *et al.*, 2008; Hõrak *et al.*, 2016). These two MPKs inhibit the protein kinase HT1 (Hashimoto *et al.*, 2006; Hõrak *et al.*, 2016; Jakobson *et al.*, 2016). Downstream of the CO_2_-specific branch are the kinases OST1 and GHR1. The inhibition of HT1 by MPK4/MPK12 releases the inhibition of OST1 and GHR1 (Hõrak *et al.*, 2016), which results in the activation of the anion channel SLAC1 (Negi *et al.*, 2008; Vahisalu *et al.*, 2008; Geiger *et al.*, 2009; Hua *et al.*, 2012) and other ion channels in the plasma and vacuolar membranes, leading to loss of turgor and stomatal closure (Kollist *et al.*, 2014; Hedrich and Geiger, 2017; Jezek and Blatt, 2017). Recent research has identified the BIG protein as an additional component of the CO_2_-specific branch of the signalling pathway. Although the exact molecular function of BIG is unknown, it was shown to induce anion currents in response to elevated HCO_3_^−^ concentration (He *et al.*, 2018). The mechanism by which changes in CO_2_ and/or HCO_3_^−^ concentration are sensed is currently unknown and it is likely that more components and interactions of the guard cell CO_2_ response pathway remain to be discovered.

The current understanding of genetic and molecular controls of the stomatal CO_2_ response is largely based on studies of the response to short-term fluctuations in CO_2_ concentration, i.e. the change in *g*_s_ that occurs within minutes to hours after a change in the atmospheric CO_2_ concentration (Vahisalu *et al.*, 2008; Engineer *et al.*, 2016). It is, however, unclear whether the short-term responsiveness is a good predictor of long-term changes in *g*_s_ of plants grown under elevated CO_2_ concentration, i.e. changes in *g*_s_ that occur over weeks to months (Morison, 1998; Haworth *et al.*, 2013). Long-term responsiveness might represent both changes in aperture and density as it entails development of new leaves. Moreover, the potential links between short- and long-term *g*_s_ responses on a molecular level have not been explored. In a synthesis of data from free air CO_2_ enrichment (FACE) experiments on trees, Hasper *et al.* (2017) observed a correlation between short-term stomatal responsiveness to changes in the CO_2_ concentration and long-term reductions in *g*_s_ of plants grown under elevated CO_2_. However, other studies have indicated that *g*_s_ may acclimate to growth under elevated CO_2_ (Šantrůček and Sage, 1996; Morison, 1998; Lodge *et al.*, 2001; Medlyn *et al.*, 2001), possibly as a result of altered stomatal sensitivity to CO_2_ (Onandia *et al.*, 2011; Haworth *et al*., 2013, 2016). In addition, short- and long-term stomatal responses may be decoupled in cases where plants respond to prolonged CO_2_ exposure by adjusting stomatal size or density rather than aperture (Haworth *et al*., 2013, 2015).

In this study, we investigated the genetic controls of both short-term (within an hour) and long-term (within a month) responses of *g*_s_ to elevated atmospheric CO_2_ concentration in *A. thaliana*. We identified genetic loci associated with short- and long-term *g*_s_ responses to elevated CO_2_, and with several other traits related to stomatal regulation. We found that a major quantitative trait locus (QTL) associated with the short-term response to CO_2_ was also involved in the long-term regulation of *g*_s_ in response to growth in elevated CO_2_ concentration. This QTL was related neither to the ABA-induced stomatal closure pathway nor to any known genetic components of stomatal regulation.

## MATERIALS AND METHODS

### Plant material and growth conditions

**Fig. 1.**
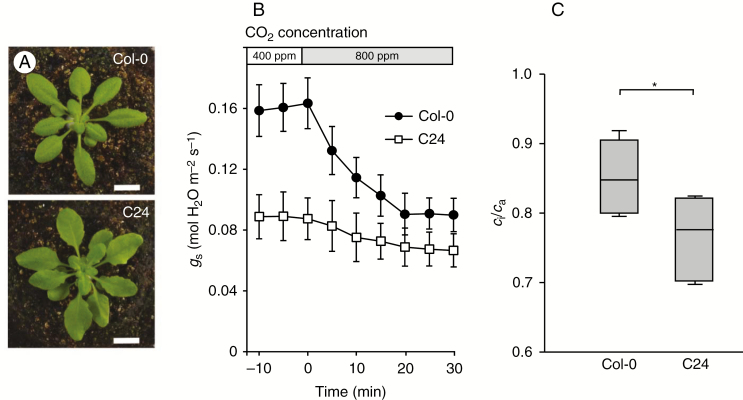
Stomata-related traits of C24 and Col-0 grown in ambient CO_2_. (A) Plants grown side by side for 26 d in ambient CO_2_ concentration, 12:12 h photoperiod and a light intensity of 150 µmol photons m^−2^ s^−1^. Scale bar = 1 cm. (B) Short-term response of *g*_s_ to elevation of CO_2_ concentration from 400 to 800 ppm during gas exchange measurements (*n* = 6, error bars show standard deviation). (C) Intrinsic water-use efficiency (iWUE) as measured by *c*_i_/*c*_a_, where low values represent high iWUE (*n* = 5). Boxes represent 25–75 % quartiles with the median as a horizontal line inside, and whiskers indicate the smallest and largest values. *P < 0.05, Welch’s *t*-test.

Recombinant inbred lines (RILs) originating from a reciprocal cross between the *Arabidopsis thaliana* accessions C24 and Col-0 (Törjék *et al.*, 2006) were used in this study. These two accessions were selected based on a pilot study of the short-term stomatal response to elevated CO_2_ concentration among various *A. thaliana* accessions, where C24 was identified as a weak responder and Col-0 as a strong responder (Fig. 1). To confirm the location of a major QTL, we additionally used reciprocal near-isogenic lines (NILs) between C24 and Col-0 (Törjék *et al.*, 2008).

The following growth conditions were used for all plants except the RILs used in ABA response measurements and the NILs used for confirmation of a major QTL: seeds were sown on soil–perlite mix, stratified at 4 °C for 2 d and cultivated under short-day conditions (8 h light/16 h darkness; 22/18 °C) at ~60 % relative humidity and a photosynthetic photon flux density of 150–170 µmol photons m^−2^ s^−1^ in growth chambers (model AR-82L2/DE, Percival Scientific, Perry, IA, USA). Seedlings were transplanted to individual pots 2 weeks after germination. In the CO_2_ experiment, plants were grown in two separate, identical growth chambers (same as above) with contrasting CO_2_ concentrations. The ambient treatment had an average daytime CO_2_ concentration of 420 ppm and CO_2_ in the elevated treatment was maintained at an average daytime concentration of 820 ppm using a TKG-CO2-3011C CO_2_ control device (Tongdy Control Technology, Beijing, China). To avoid confounding effects of between- and within-chamber variation in environmental conditions, plants and CO_2_ treatment levels were shifted between the two growth chambers twice a week and trays with pots were rotated 180 °C.

Seeds used to generate plants for ABA response measurements and for confirmation of a major QTL were stratified in water for 2 d at 4 °C, sown on peat–vermiculite mix and grown through a hole in a glass plate covering the pot as described previously (Kollist *et al.*, 2007) under short-day conditions (12 h light/12 h darkness, 23/20 °C) at 70 % relative humidity and a light intensity of 100–150 μmol m^−2^ s^−1^ in growth chambers (Microclima Arabidopsis MCA1600-3LP6-E, Snijders Scientific, Tilburg, the Netherlands).

### Study design

**Fig. 2.**
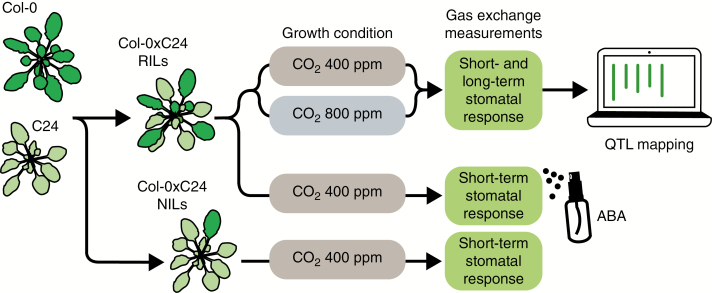
Schematic overview of the experimental setup. *Arabidopsis* wild-type parental accessions Col-0 and C24, and recombinant inbred lines (RILs) and near-isogenic lines (NILs) originating from crosses between these accessions, were cultivated in the indicated conditions and subjected to measurement of gas exchange and carbon isotope ratios (not shown). Phenotypic data of RILs combined with genotype data of RILs were used for QTL mapping. Short-term stomatal response refers to a change in stomatal conductance within hours after the application of a stimulus, whereas the long-term response corresponds to the treatment effect when plants were grown in two different CO_2_ concentrations and measured at their respective growth concentration. The stomatal response to ABA was measured in a subset of RILs displaying the most extreme CO_2_ response phenotypes, to investigate whether a major CO_2_ response QTL was involved in the CO_2_-specific pathway for stomatal closure or in the downstream signalling pathway where CO_2_ and ABA responses converge. The short-term CO_2_ response of NILs was used to confirm the presence, location and effect of this major QTL.

The study comprised three experiments to investigate various aspects of stomatal regulation (as illustrated in Fig. 2).

#### QTL mapping of plants grown in ambient CO_2_

The initial QTL mapping experiment was designed to identify genetic loci associated with the short-term (within minutes to hours) response of *g*_s_ to elevated CO_2_, with absolute *g*_s_ at ambient and elevated CO_2_, and with the ratio of mole fractions of CO_2_ in the substomatal cavity and ambient air, *c*_i_/*c*_a_. The latter is a proxy for intrinsic water-use efficiency (iWUE), where low values represent high iWUE. We selected a subset of 100 RILs that displayed the largest number of chromosomal crossovers in the population, in order to maximize genetic variation (Supplementary Data Table S1). Of these RILs, 51 originated from a cross using C24 as pollen donor and Col-0 as pollen acceptor, and 49 where Col-0 was used as pollen donor to C24, to account for potential cytoplasmic effects. The RILs and their parental accessions were grown at ambient CO_2_ concentration. The short-term CO_2_ response, absolute *g*_s_, and *c*_i_/*c*_a_ were quantified and these data were used for QTL mapping. Fine mapping of a major QTL controlling the short-term *g*_s_ response to elevated CO_2_ was performed using additional RILs with crossovers in the region of interest and the location and the effect of the QTL was confirmed using NILs.

#### Long-term CO_2_ experiment

The aim of this experiment was to study the effects of growth in elevated CO_2_ on stomatal regulation in *A. thaliana*. Specifically, we wanted to (1) investigate whether growth in elevated CO_2_ concentration affected the short-term (within minutes to hours) CO_2_ responsiveness and absolute *g*_s_, as well as the detection of QTLs associated with CO_2_ responsiveness, and (2) map loci associated with the long-term (within weeks to months) response to elevated CO_2_. We used 50 RILs from the cross where C24 was the pollen donor and Col-0 the acceptor, which had been used in the previous experiment. The RILs were grown together with their parental accessions in ambient or elevated CO_2_ in two separate treatments for 4 weeks, which constitutes a large proportion of the *A. thaliana* life cycle (Boyes *et al.*, 2001). Data on short-term CO_2_ response and absolute *g*_s_ of plants from both CO_2_ treatments were used for QTL mapping, as well as data on the long-term *g*_s_ response to elevated CO_2_.

#### ABA experiment.

The stomatal response to exogenously applied ABA was measured in ten RILs that showed the five strongest and the five weakest CO_2_ responses in the first experiment. The aim of the ABA experiment was to investigate the relationship between CO_2_- and ABA-induced stomatal closure in these lines.

### Gas exchange measurements

In the first two experiments, gas exchange measurements on entire leaf rosettes of 4-week-old plants were conducted using two LI6400 systems (LI-COR Biosciences, Lincoln, NE, USA) fitted with 6400-17 Whole Plant Arabidopsis Chambers. Self-shading within rosettes was minimal at this growth stage. Leaf temperature was estimated using energy balance calculations (LI-COR Biosciences, 2011). The boundary layer conductance was estimated using a model of a leaf rosette made from filter paper, which was soaked in water that was allowed to evaporate inside the whole plant chamber. The boundary layer conductance was estimated to be 4 mol H_2_O m^−2^ s^−1^. The stomatal ratio (the ratio of *g*_s_ on the leaf sides with lowest versus highest values) was assumed to be 0.5, which is recommended when the exact ratio is not known (LI-COR Biosciences, 2011). Fluxes of water vapour from the soil were prevented by covering the soil using household cling film. To test for the influence of water vapour exiting through the tiny gap in the plastic film surrounding the stem, we conducted measurements after cutting the plant rosette above the plastic film. The false conductance measured was <0.002 mol H_2_O m^−2^ s^−1^ (assuming a leaf area of 10 cm^2^) and was considered negligible.

Gas exchange measurements in the two first experiments were conducted at constant light (same as growth conditions) and temperature (22 °C). The vapour pressure deficit (VPD) of the air was set to a target value within the range 1 ± 0.2 kPa and was kept within ±0.03 kPa of the initial VPD throughout the measurement of a plant. The CO_2_ concentration was kept at 400 ppm until *g*_s_ reached steady state (<2.5 % change in conductance over 5 min). When steady state had been reached, three measurements with 10 s between them were logged, after which the CO_2_ concentration was elevated to 800 ppm and the same procedure was repeated. Plant leaves were imaged using a flatbed scanner, leaf areas were calculated using the ROI manager function in ImageJ (version 1.48v, Schneider *et al.*, 2012) and the conductance values were re-calculated to be expressed per unit leaf area. The percentage reduction in *g*_s_ following elevation of the CO_2_ concentration was used as a measure of the short-term stomatal response to elevated CO_2_. In the CO_2_ experiment, we additionally calculated the long-term response to growth in elevated CO_2_ concentration. For the same genotype, we used *g*_s_ values of plants from the two treatments measured at their respective growth CO_2_ concentration to calculate the percentage decrease in stomatal conductance resulting from growth in elevated CO_2_.

For the ABA response experiment and for the confirmation of a major QTL using NILs, gas exchange measurements were conducted on 25- to 30-d-old plants using a custom-made gas exchange system (Kollist *et al.*, 2007). We quantified the percentage *g*_s_ decrease in response to elevated CO_2_ (~800 ppm) in both experiments and in the ABA experiment also to spray application of 5 µm ABA solution (containing 0.012 % Silwet and 0.05 % ethanol). Measurements were conducted at a light intensity of 100–150 µmol photons m^−2^ s^−1^ and a temperature of 23–25 °C. Stomatal conductance was allowed to stabilize at ambient CO_2_ concentration and 65–70 % relative humidity for ~40 min before the stimulus was applied. The stomatal response was calculated as the percentage *g*_s_ decrease 28 min after application of the stimulus. Leaf areas were measured using the polygon tool in ImageJ (version 1.48v, Schneider *et al.*, 2012) on photographs of intact leaf rosettes.

### Stable isotope analyses

The ratio of mole fractions of CO_2_ in the substomatal cavity (*c*_i_) and ambient air (*c*_a_), *c*_i_/*c*_a_, was used as a proxy for iWUE, where low *c*_i_/*c*_a_ corresponds to a high iWUE (Condon *et al.*, 2004; Pérez-Harguindeguy *et al.*, 2013) as shown by the relationship:

$$\begin{array}{l} \frac{c_{i}}{c_{a}}=1-\frac{iWUE\times 1.6}{c_{a}} \end{array}$$

A time-integrated measure of *c*_i_/*c*_a_ was determined by analysing leaf stable carbon isotope composition. Leaves were dried for at least 24 h at 70 °C and homogenized with a pestle. The material (~1 mg per sample) was weighed into tin capsules and analysed for stable carbon isotope ratios using a PDZ Europa ANCA-GSL elemental analyser interfaced to a PDZ Europa 20-20 isotope ratio mass spectrometer (IRMS; Sercon, Crewe, UK) at the UC Davis Stable Isotope Facility, Davis, CA, USA. The photosynthetic ^13^C discrimination (Δ) was calculated from δ ^13^C values according to the following equation:

$$\begin{array}{l} \Delta =\frac{\delta^{13}C_{air}-\delta^{13}C_{plant}}{1+\delta^{13}C_{plant}} \end{array}$$

For this calculation we assumed a value of −8.44 ‰ for δ ^13^C_air_, based on the average δ ^13^C ratio of CO_2_ in air measured at the Mauna Loa Observatory, HI, USA during 2014 (data downloaded from https://www.esrl.noaa.gov, Keeling *et al.*, 2001). This value likely differed slightly from that in our experiment, but as the isotope data were only used to compare plants within the same experiment this error was considered negligible. The ^13^C discrimination was then used for the calculation of *c*_i_/*c*_a_ as follows:

$$\begin{array}{l} \frac{c_{i}}{c_{a}}=\frac{\Delta -a}{b-a} \end{array}$$

where *a* is the isotopic fractionation caused by diffusion (4.4 ‰) and *b* is the fractionation caused by carboxylation by Rubisco (27 ‰) (Farquhar *et al.*, 1989). It should be noted that the above equations follow the simplified format presented by Farquhar *et al.* (1989), where ‰ is considered equivalent to 10^–3^; hence, all ‰ values were multiplied by 0.001 in our calculations.

### Genotyping

The RIL population had previously been genotyped using SNP markers, as described by Törjék *et al.* (2003). The lines used in this study were partially re-genotyped in generations *F*_9_/*F*_10_ to confirm or correct double crossovers and to remove heterozygous regions. For this purpose, the SNaPshot^®^ Multiplex System (Applied Biosystems, Waltham, MA, USA) was used according to the manufacturer’s protocol on an ABI 3730 Sequencer (Applied Biosystems). Peaks were identified using GeneMapper^®^ (version 4.0, Applied Biosystems). In addition, simple sequence length polymorphism (SSLP) markers from the MSAT database (http://www7.inra.fr/vast/msat.php) were added to allow comparison with other *A. thaliana* RIL populations and single-feature polymorphism (SFP) markers were extracted from ATH1 GeneChip^®^ (Affymetrix, Waltham, MA, USA) data as described by Schmidt *et al.* (2017). The SSLP fragments were PCR-amplified from genomic DNA and visualized on agarose gels. For large fragments and/or size differences above 10 bp, 1–2 % agarose gels (Carl Roth, Karlsruhe, Germany) were used. For smaller size differences, a 1:3 mixture of 4 % agarose/MetaPhor™ agarose (Lonza Group, Basel, Switzerland) was used. Fragment size was identified by comparison with the Generuler™ 1 kb Plus DNA Ladder (Thermo Fisher Scientific, Waltham, MA, USA) and genotypes were scored manually. Genetic maps for the two subsets of lines analysed in this study were constructed using the package R/qtl (version 1.41-6) in R (version 3.4.3) with the Kosambi mapping function (Broman *et al.*, 2003). The NILs had been genotyped using the same set of SNP markers as the RILs, as described by Törjék *et al.* (2008).

For fine mapping of the major QTL on chromosome 2, 42 RILs with crossovers in the region of interest were used. Genotyping was performed using nine SSLP markers (Supplementary Data Table S2) in the QTL region following the methodology of Nilsson *et al.* (2016). Annealing temperatures were optimized for each primer pair using gradient PCR. PCR products were visualized on 3 % agarose gels (Seakem^®^ LE, Lonza Group, Basel, Switzerland) and genotypes scored manually.

In order to confirm the lack of sequence variation in the *MPK12* gene between C24 and Col-0 in publicly available sequence data (Berardini *et al.*, 2015; Alonso-Blanco *et al.*, 2016), a genomic fragment consisting of the coding region and 0.5-kb flanking region on both sides was PCR amplified from C24 using three sets of primers (Supplementary Data Table S2) and AccuPrime™ *Pfx* polymerase (Thermo Fisher Scientific, Waltham, MA, USA) to produce three overlapping fragments. The products were sequenced (Eurofins Genomics, Ebersberg, Germany) using the same set of primers, with an additional sequencing primer for one of the products (Supplementary Data Table S2).

### Data analysis

Differences in the short-term CO_2_ response and absolute *g*_s_ of accessions C24 and Col-0 were tested using Welch’s *t*-test. Welch’s *t*-test was also used to test for cytoplasmic effects by comparing trait averages from the reciprocal crosses. Differences in short-term response and absolute *g*_s_ between RILs grown in ambient and elevated CO_2_ treatments were tested using the paired *t*-test. The relationship between short- and long-term stomatal CO_2_ responses was tested using linear regression and the paired *t*-test was used to test for a difference in magnitude of these responses. One-way ANOVA with Tukey’s *post hoc* test was used to test for differences between NILs and parental accessions. All statistical tests were performed with *α* = 0.05 using JMP (version 12.0.1, SAS Institute, Cary, NC, USA).

For QTL mapping, data from both crosses were analysed together as no significant cytoplasmic effect on any of the traits had been detected. Data from the first experiment, where 100 RILs were grown in ambient CO_2_, and the second experiment, where 50 RILs were grown in two CO_2_ treatments (ambient and elevated), were analysed separately. To increase mapping power and enable the identification of QTLs with pleiotropic effects, we used multi-trait analysis combining all phenotype data from each experiment. A step size of 10 cM, minimum cofactor proximity of 50 cM, a minimum separation of selected QTLs of 30 cM and a threshold of –log10P = 3.2 (based on Li and Ji, 2005) were used for QTL analysis. First, the whole genome was scanned for significant polymorphisms using simple interval mapping. Then, based on the selected cofactors, two rounds of composite interval mapping were run. Thereafter, a final QTL model was selected using backward selection on the selected cofactors, where the allelic effect and explained phenotypic variance of each QTL were estimated for each trait. All QTL analyses were performed in GenStat for Windows (16th edition, VSN International, Hemel Hempstead, UK).

## RESULTS

### Stomatal regulation of parental accessions

We observed significantly weaker short-term stomatal CO_2_ response, i.e. the percentage decrease in stomatal conductance (*g*_s_) following a doubling of the CO_2_ concentration (Welch’s *t*-test, *P* = 0.01, *n* = 6), as well as lower absolute *g*_s_ at both 400 and 800 ppm CO_2_ of C24 compared with Col-0 (Welch’s *t*-test, *P* < 0.001 and *P* = 0.002, respectively, *n* = 6; Fig. 1B), confirming the results of the pilot study. Furthermore, C24 demonstrated a significantly lower *c*_i_/*c*_a_ than Col-0, showing that C24 had a higher intrinsic water-use efficiency (iWUE) than Col-0 (Welch’s *t*-test, *P* = 0.043, *n* = 5; Fig. 1C). In summary, C24 generally has lower stomatal conductance and thus a more conservative regulation of transpirational water loss but at the same time its stomata are less responsive to increased CO_2_ concentration.

### QTL mapping of stomatal regulation in 100 RILs grown in ambient CO_2_

**Fig. 3.**
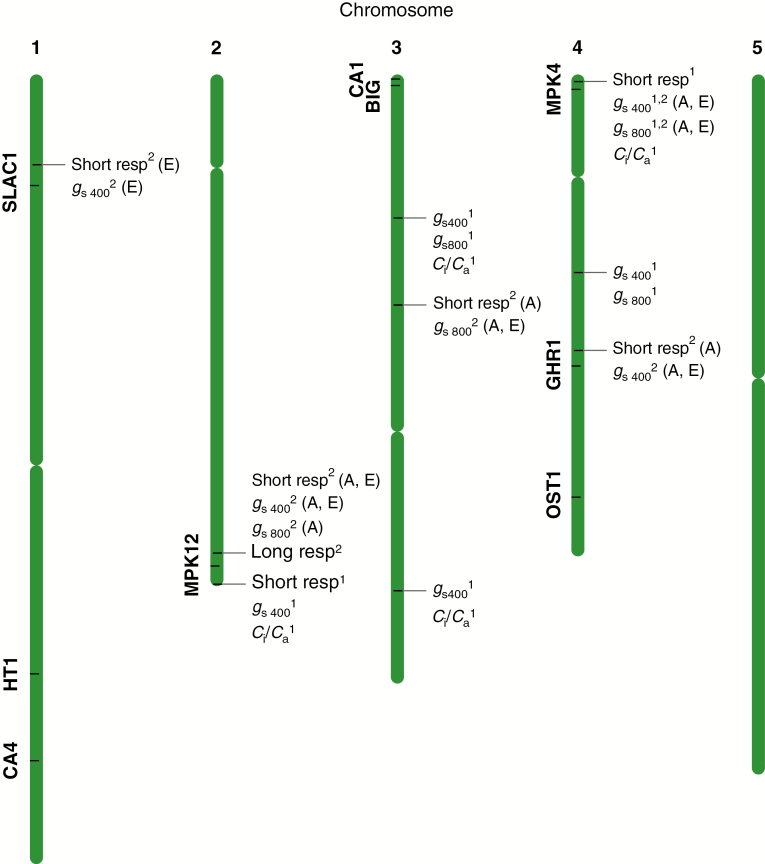
Chromosomal positions of all QTLs detected in this study and of known components of the stomatal CO_2_ response pathway. ^1^Detected in the first experiment with 100 recombinant inbred lines (RILs) grown in ambient CO_2_ concentration. ^2^Detected in the second experiment with 50 RILs grown in two CO_2_ treatments: ambient (A) and elevated (E) CO_2_. Short resp, short-term response; Long resp, long-term response.

To investigate the genetic basis for the variation in stomatal regulation between Col-0 and C24, we quantified several stomata-related traits among 100 RILs originating from a reciprocal cross between these accessions and used these data for QTL mapping. The short-term *g*_s_ response to elevated CO_2_ ranged from 13 to 64 % among the RILs (Supplementary Data Fig. S1, Table S3). The RILs also displayed a wide range in absolute *g*_s_ (*g*_s_400_ 0.062–0.174 mol m^−2^ s^−1^, *g*_s_800_ 0.038–0.122 mol m^−2^ s^−1^) and *c*_i_/*c*_a_ (0.668–0.905) (Supplementary Data Fig. S1, Tables S3 and S4). We detected a major QTL for the short-term CO_2_ response on chromosome 2, explaining 51 % of the variation in this trait. The Col-0 allele at this QTL conferred a stronger CO_2_ response (Table 1). An additional, minor QTL (explaining 3 % of the variation) for the short-term CO_2_ response was mapped to chromosome 4 (Fig. 3, Table 1). For this QTL, C24 was instead the high-value allele (Table 1).

**Table 1.** QTLs detected using data from measurements of gas exchange and stable isotope composition of recombinant inbred lines grown in ambient CO_2_

Trait	QTL location and confidence interval (cM)	Chromo-some	Closest marker	Variance explained (%)	High-value allele
Short-term CO_2_ response	110 (105–110)	2	MASC02812/MSAT2.22	51	Col-0
*g*_s_400_	110 (105–110)	2	MASC02812/MSAT2.22	19	Col-0
*c*_i_/*c*_a_	110 (105–110)	2	MASC02812/MSAT2.22	13	Col-0
*g*_s_400_	23 (0–108)	3	MASC04608	4	Col-0
*g*_s_800_	23 (0–108)	3	MASC04608	9	Col-0
*c*_i_/*c*_a_	23 (0–108)	3	MASC04608	9	Col-0
*g*_s_400_	94 (0–108)	3	MASC03218	6	Col-0
*c*_i_/*c*_a_	94 (0–108)	3	MASC03218	4	Col-0
Short-term CO_2_ response	5 (0–35)	4	FRI	3	C24
*g*_s_400_	5 (0–35)	4	FRI	3	Col-0
*g*_s_800_	5 (0–35)	4	FRI	14	Col-0
*c*_i_/*c*_a_	5 (0–35)	4	FRI	4	Col-0
*g*_s_400_	52 (6–92)	4	MASC09213	7	C24
*g*_s_800_	52 (6–92)	4	MASC09213	12	C24

Five QTLs related to absolute *g*_s_ were detected, of which three were associated with *g*_s_ at both 400 and 800 ppm CO_2_, and two were associated with *g*_s_ only at 400 ppm (Fig. 3, Table 1). The amount of variation explained by these QTLs ranged from 3 to 19 %. Notably, the strongest QTL for *g*_s_ measured at 400 ppm mapped to the same marker as the short-term CO_2_ response. The high-value allele was Col-0 for most of the QTLs regulating *g*_s_ (Table 1), consistent with the higher *g*_s_ of the Col-0 parental accession. Furthermore, four QTLs for *c*_i_/*c*_a_ were detected (Fig. 3, Table 1). These QTLs explained 4–13 % of the variation in this trait and the high-value allele was Col-0 in all cases, consistent with the observation of lower iWUE in Col-0. The strongest QTL for *c*_i_/*c*_a_ also mapped to the same locus as the strongest QTL for short-term CO_2_ response (Table 1).

### Effects of long-term growth in elevated CO_2_

We next sought to investigate the relationship between the control of short- and long-term *g*_s_ responses. To this end, 50 RILs from the cross where C24 was the pollen donor and Col-0 the pollen acceptor, which had been used in the previous experiment, were cultivated in ambient (~400 ppm) and elevated (~800 ppm) CO_2_ concentrations. After 4 weeks of the respective treatment, gas exchange measurements were conducted. The short-term response was calculated as the percentage decrease in *g*_s_ between 400 and 800 ppm measured in sequence for each individual. The long-term response was calculated as the percentage decrease in *g*_s_ resulting from growth in elevated CO_2_, i.e. for the same genotype we used *g*_s_ values of plants from the two treatments measured at their respective growth CO_2_ concentration.

**Fig. 4.**
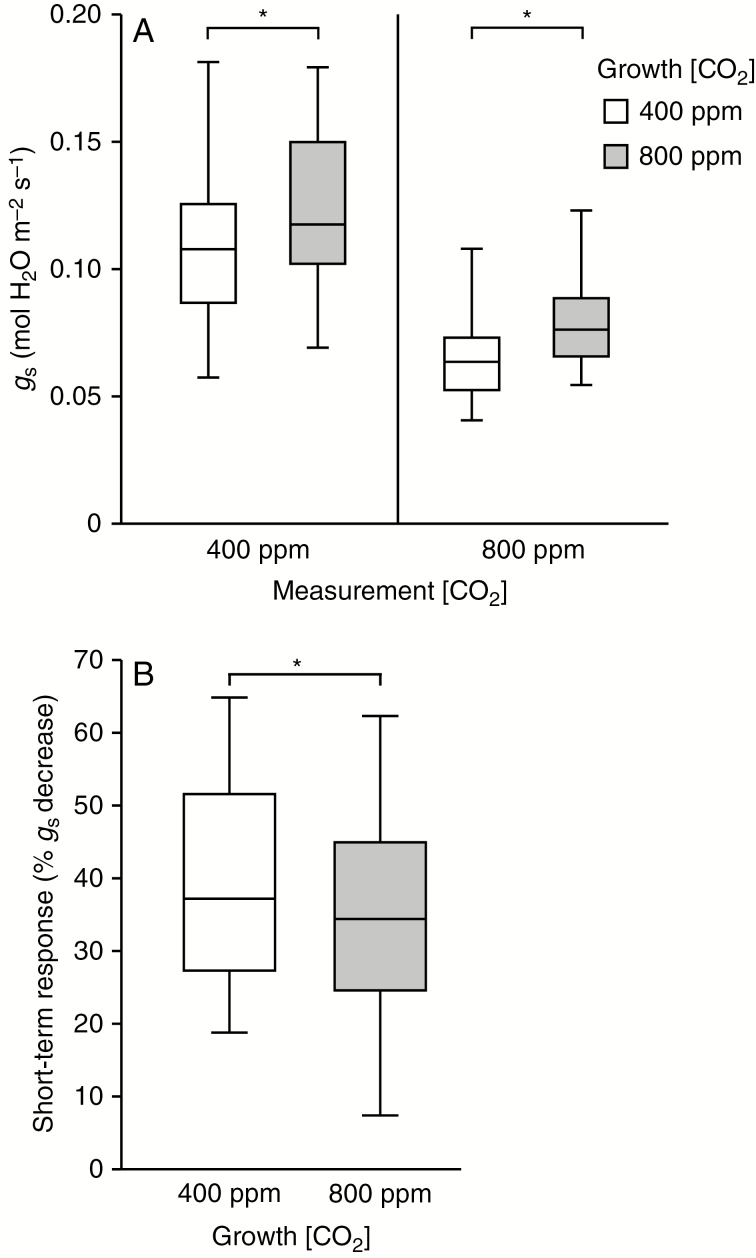
Stomatal conductance (A) and short-term CO_2_ response (B) of 50 recombinant inbred lines grown in ambient and elevated CO_2_. Gas exchange was measured at 400 and 800 ppm CO_2_ in sequence. The short-term CO_2_ response was calculated as the percentage decrease in *g*_s_ after elevation of the CO_2_ concentration from 400 to 800 ppm during gas exchange measurements. Boxes represent 25–75 % quartiles with the median as a horizontal line inside, and whiskers indicate the smallest and largest values. *P < 0.05, paired *t*-test (*n* = 50); note that the significant long-term response, i.e. paired t-test comparing gs of plants from the two CO2 treatments when measured at their respective growth CO2 concentration (*P* < 0.0001, *n* = 50), is not indicated in panel (A).

Among the tested RILs, growth in elevated CO_2_ concentration resulted in an average *g*_s_ reduction of 26 % (paired *t*-test, *P* < 0.0001, *n* = 50; Fig. 4A, Supplementary Data Table S4). When the *g*_s_ of plants grown in ambient and elevated CO_2_ was measured at the same CO_2_ concentration, plants from the elevated treatment generally displayed higher *g*_s_ than plants from the ambient treatment. On average, plants from the elevated treatment had 11 % higher *g*_s_ than plants from the ambient treatment when measured at 400 ppm and 20 % higher *g*_s_ when measured at 800 ppm (paired *t*-test of *g*_s_400_, *P* = 0.004, *n* = 50; paired *t*-test of *g*_s_800_, *P* < 0.0001, *n* = 50; Fig. 4A, Supplementary Data Table S4), indicating *g*_s_ acclimation of plants grown in elevated CO_2_.

Growth under elevated CO_2_ concentration had a small but statistically significant effect on the short-term CO_2_ response (paired *t*-test, *P* = 0.0004, *n* = 50). RILs grown in ambient CO_2_ concentration showed an average *g*_s_ decrease of 39 % in response to short-term elevation of the CO_2_ concentration, whereas the average short-term *g*_s_ response of RILs grown in elevated CO_2_ was 34 % (Fig. 4B, Supplementary Data Table S4), indicative of a slight decrease in CO_2_ sensitivity of plants grown in elevated CO_2_.

**Fig. 5.**
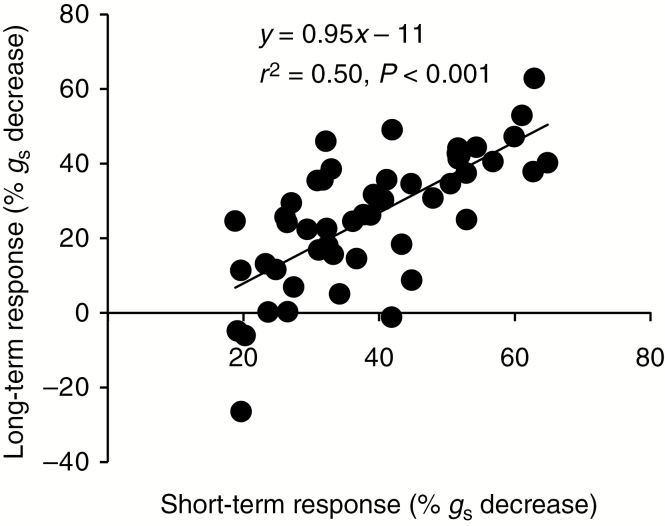
Long-term versus short-term stomatal response to elevated CO_2_ of 50 recombinant inbred lines cultivated in ambient or elevated CO_2_. Plants were grown in the respective CO_2_ treatments for 4 weeks and gas exchange was measured for each line and treatment at 400 and 800 ppm CO_2_ in sequence. The short-term CO_2_ response of each line was calculated as the percentage decrease in *g*_s_ upon short-term elevation of the CO_2_ concentration of plants grown in ambient CO_2_. The long-term response of each line represents the percentage *g*_s_ decrease in the elevated compared with the ambient treatment when plants were measured at their respective growth CO_2_ concentration. There was a significant linear relationship between the long- and short-term responses, but the long-term response was significantly weaker than the short-term response (paired *t*-test, *P* < 0.001, *n* = 50).

To test whether the short-term response could be used as a predictor of the long-term response, the short-term response of each genotype grown in ambient CO_2_ concentration was compared with the long-term response of the same genotype (Fig. 5). This showed a significant linear relationship between the responses (linear regression *P* < 0.0001, *r*^2^ = 0.53, *F*_1,48_ = 48.4). The long-term response was, however, significantly weaker than the short-term response (26 % versus 39 % *g*_s_ decrease; paired *t*-test, *P* < 0.001, *n* = 50).

### QTL mapping of stomatal regulation following long-term cultivation in elevated CO_2_

Data on short- and long-term stomatal CO_2_ responses and absolute *g*_s_ from plants grown in two CO_2_ treatments were used for QTL mapping. The major QTL on chromosome 2 associated with the short-term CO_2_ response, identified in the previous experiment on plants grown in ambient CO_2_, was detected in plants grown in both ambient and elevated CO_2_. Using the subset of 50 RILs, this QTL mapped to the adjacent marker compared with the results from the previous experiment (Fig. 3, Table 2). Additionally, three other, minor QTLs for the short-term response were identified, explaining 7–10 % of the variation in this trait. These QTLs were detected only in one of the CO_2_ treatments (Table 2). For the long-term *g*_s_ response to elevated CO_2_, one QTL explaining 14 % of the variation was identified (Fig. 3, Table 2). This QTL mapped to the same marker as the major QTL for the short-term response, suggesting that these traits are regulated by the same genetic component. Furthermore, five QTLs for absolute *g*_s_ were detected, of which two were associated with *g*_s_ measured at both 400 and 800 ppm, two with *g*_s_ at 400 ppm and one with *g*_s_ at 800 ppm. Most QTLs for absolute *g*_s_ were detected in plants from both CO_2_ treatments (Fig. 3, Table 2).

**Table 2.** QTLs detected using data from gas exchange measurements on recombinant inbred lines grown in ambient (A) or elevated (E) CO_2_ concentration

Trait	QTL location and confidence interval (cM)	Chromo-some	Closest marker	Treatment	Variance explained (%)	High-value allele
Short-term CO_2_ response	18 (0–138)	1	MASC09203	E	8	C24
*g*_s_400_	18 (0–138)	1	MASC09203	E	13	C24
Short-term CO_2_ response	123 (104–125)	2	MASC00371	A and E	A 36 E 27	Col-0
*g*_s_400_	123 (104–125)	2	MASC00371	A and E	A 8 E 12	Col-0
*g*_s_800_	123 (104–125)	2	MASC00371	A	8	C24
Long-term CO_2_ response	123 (104–125)	2	MASC00371	–	14	Col-0
Short-term CO_2_ response	38 (0–101)	3	MSAT3.19/MASC04516	A	7	C24
*g*_s_800_	38 (0–101)	3	MSAT3.19/MASC04516	A and E	A 18 E 9	Col-0
*g*_s_400_	6 (0–42)	4	FRI/MASC04123	A and E	A 11 E 16	Col-0
*g*_s_800_	6 (0–42)	4	FRI/MASC04123	A and E	A 9 E 25	Col-0
Short-term CO_2_ response	79 (0–103)	4	MASC02548/F24J7ID/ G3883-1.4	A	10	C24
*g*_s_400_	79 (0–103)	4	MASC02548/F24J7ID/ G3883-1.4	A and E	A 15 E 8	C24

### ABA response

**Fig. 6.**
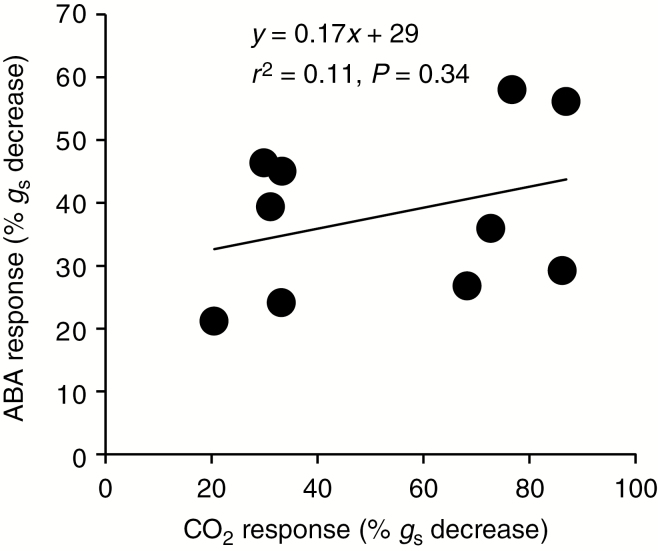
There was no significant relationship between stomatal responses to exogenous abscisic acid (ABA) and elevated CO_2_. Five recombinant inbred lines displaying the weakest and five displaying the strongest CO_2_ responses were sprayed with 5 µm ABA. The percentage decrease in *g*_s_ following application was quantified using gas exchange measurements. Measurements were performed on two or three replicates per line.

As the signalling pathway for the CO_2_-induced closure response is known to converge downstream with the pathway for ABA-induced stomatal closure, we tested whether the main loci involved in CO_2_-induced stomatal closure also affected the ABA-induced stomatal response. To this end, ten RILs representing contrasting genetic backgrounds and CO_2_-induced closure phenotypes were cultivated at ambient CO_2_ concentration and stomatal conductance was monitored after spray application of ABA. Measurements were performed on two or three replicates per line. This showed that the ability to close stomata in response to exogenous ABA was not correlated with the ability to respond to CO_2_ (Fig. 6), suggesting that the QTL for stomatal CO_2_ response identified in this study is not involved in ABA-induced stomatal closure.

### Fine mapping of a locus on chromosome 2 controlling CO_2_-induced closure

The major QTL associated with CO_2_-induced stomatal closure among the RILs mapped to the end of chromosome 2. To narrow down the region of interest, nine new SSLP markers (Supplementary Data Table S2) spanning the area between markers MASC06025 and MASC02812 were developed and used to genotype a subset of RILs with crossovers at the end of chromosome 2. This approach narrowed the region to a physical distance of 410 kb between markers MASC02812 and MASC00371 (Supplementary Data Table S5), consistent with previous mapping results that located this QTL to either of these two markers depending on the subset of RILs. This region contains the *MPK12* gene, which encodes a kinase recently shown to be involved in CO_2_-induced stomatal closure (Hõrak *et al.*, 2016; Jakobson *et al.*, 2016). However, neither publicly available sequence data (Berardini *et al.*, 2015; Alonso-Blanco *et al.*, 2016) nor results from our own sequencing show any sequence differences between C24 and Col-0 for this gene or 0.5-kb flanking regions, except for a single SNP 0.47 kb downstream of the coding sequence.

### Confirmation of the major QTL on chromosome 2 using NILs

**Fig. 7.**
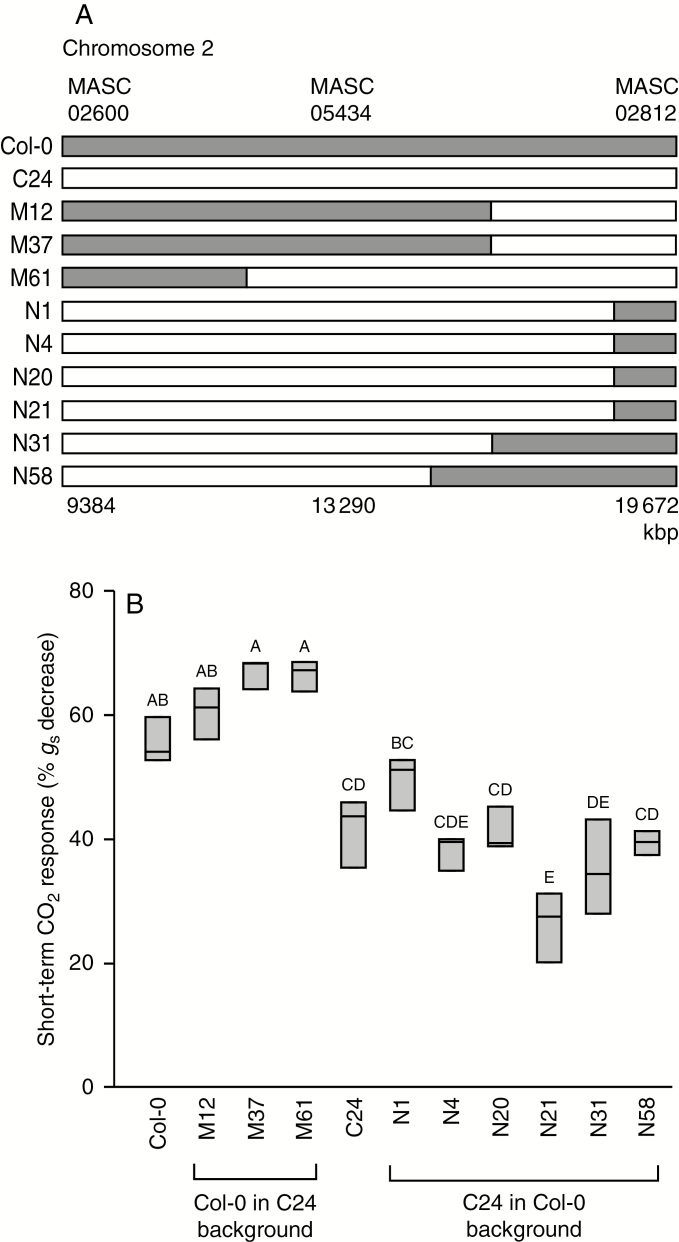
Genotype information (A) and short-term stomatal CO_2_ response (B) of the parental accessions (Col-0 and C24) and nine reciprocal near-isogenic lines with introgressions at the end of chromosome 2, i.e. in the region of a major QTL associated with the short-term CO_2_ response, which was identified by QTL mapping using recombinant inbred lines. Boxes represent 25–75 % quartiles with the median as a horizontal line inside, and whiskers indicate the smallest and largest values. Different letters indicate a statistically significant difference in Tukey’s *post hoc* test (*P* < 0.05, *n* = 3).

The short-term CO_2_ response of nine NILs (Supplementary Data Table S1; Törjék *et al.*, 2008) measured in triplicate was used to confirm the location and effect of the major QTL identified and characterized in previous experiments with RILs. In these lines, the genome was predominantly from one of the parents, with a small introgression of the opposite genotype at the end of chromosome 2. Three lines were on the C24 background and six on the Col-0 background (Fig. 7A). Lines on the Col-0 background with introgression from C24 changed their CO_2_ response to the C24 phenotype and vice versa; lines on the C24 background with Col-0 introgression gained the Col-0 phenotype (Fig. 7B). One line (NIL number N1) could not be statistically distinguished from either of the parental accessions (Fig. 7B). The results of measurements of these independent lines confirm the location of the QTL between the two last markers on chromosome 2. Furthermore, these results show that the effect of the QTL was large enough to shift the phenotype from that of the background accession to one similar to the phenotype of the introgressed accession.

## DISCUSSION

In this study, we used natural variation in stomatal regulation between the two *A. thaliana* accessions C24 and Col-0 to identify genetic loci associated with short- and long-term responses of *g*_s_ to elevated CO_2_ concentration, as well as several other stomata-related traits. The short-term response represents the adjustment of *g*_s_ that occurs within minutes to hours after change in atmospheric CO_2_ concentration, whereas the long-term response represents a change in *g*_s_ seen after weeks to months of cultivation under elevated CO_2_ concentration. The use of RILs originating from a cross between C24 and Col-0 enabled the identification of a number of QTLs associated with stomatal regulation. Most notable was a QTL at the end of chromosome 2 explaining ~50 % of the variation in the short-term *g*_s_ response to elevated CO_2_ concentration among the tested RILs. Interestingly, this QTL was also associated with the long-term *g*_s_ response to growth under elevated CO_2_ concentration, suggesting that these traits are regulated by the same underlying gene. The same QTL was additionally associated with absolute *g*_s_ at ambient CO_2_ concentration and water-use efficiency. The Col-0 genotype at this locus conferred stronger CO_2_ responsiveness in both the short and the long term, as well as higher *g*_s_ at 400 ppm CO_2_. The C24 genotype was associated with higher water-use efficiency.

Exogenous application of ABA to a subset of RILs with the most extreme CO_2_ response phenotypes showed that there was no correlation between the stomatal closure responses to ABA and CO_2_. This implies that the identified major QTL is involved in the CO_2_-specific branch of the signalling pathway for stomatal closure, upstream of the convergence point for CO_2_- and ABA-induced responses. Analysis of short-term responsiveness to elevated CO_2_ in reciprocal NILs between C24 and Col-0 confirmed the location of the QTL at the end of chromosome 2. Introgressions in this region caused a significant change in responsiveness and shifted the phenotype to one similar to that of the introgressed parent. Fine mapping using RILs with crossovers in the region of interest allowed us to locate the QTL to a 410-kb region. This region contains the *MPK12* gene, which was recently shown to have a pivotal role in CO_2_-induced stomatal closure (Jakobson *et al.*, 2016). However, no sequence polymorphisms were found between C24 and Col-0 in *MPK12* that could explain the phenotypic difference. The phenotype of C24, i.e. weak CO_2_ response in combination with low *g*_s_, also differs from the phenotype resulting from known loss-of-function mutations in *MPK12*, i.e. weak CO_2_ response in combination with very high *g*_s_ (Jakobson *et al.*, 2016; Tõldsepp *et al.*, 2018). Finally, C24 and Col-0 show only a moderate difference in expression of *MPK12* (60 % higher in C24; Xu *et al.*, 2015). Taking these results together, it is thus unlikely that any polymorphism affecting the expression of *MPK12* could explain the difference in CO_2_-dependent stomatal closure between C24 and Col-0. Besides *MPK12*, the mapped region does not contain any genes previously linked to stomatal behaviour. Further studies are required to accurately pinpoint the exact molecular difference underlying this QTL. Nevertheless, our data point to the presence of at least one gene in this region that encodes an important as yet unidentified component regulating *g*_s_ and its response to elevated CO_2_ in both the short and the long term.

The fact that short- and long-term CO_2_ responses were significantly correlated and associated with the same locus suggests that knowledge about the signalling pathway for short-term *g*_s_ regulation in response to elevated CO_2_ concentration could be used for manipulation of long-term *g*_s_ responses under rising atmospheric CO_2_. Results from experimental field research corroborate the link between short- and long-term *g*_s_ responses (Hasper *et al.*, 2017), indicating that the short-term CO_2_ response may be a useful predictor of the long-term CO_2_ effect on *g*_s_ also under ecologically realistic conditions. Data on short-term responsiveness among plant species and/or varieties could thus be valuable for projections of plant water use under rising atmospheric CO_2_ concentration. Cases where short-and long-term responses are decoupled due to a pronounced stomatal density response in plants with weak short-term responsiveness (Haworth *et al.*, 2013) should, however, be a focus of further studies.

Growth under elevated CO_2_ concentration resulted in an average *g*_s_ decrease of 26 % among the tested RILs, which is similar to the average long-term response observed in field experiments (21 %, Medlyn *et al.*, 2001; 22 %, Ainsworth and Rogers, 2007). Plants grown under elevated CO_2_ exhibited slightly attenuated short-term responsiveness to CO_2_ and higher *g*_s_ compared with plants grown under ambient CO_2_ when measured at the same CO_2_ concentration. These results show that both guard cell CO_2_ responsiveness and absolute *g*_s_ acclimated to the CO_2_ concentration during growth. Previous research has shown that guard cells of plants grown under elevated CO_2_ may lose some of their sensitivity to short-term changes in CO_2_ concentration (Morison, 1998; Lodge *et al.*, 2001; Medlyn *et al.*, 2001; Onandia *et al.*, 2011). The direction of *g*_s_ acclimation in our study differed from previous observations. A meta-analysis on trees subjected to long-term CO_2_ exposure showed that photosynthetic capacity and *g*_s_ were downregulated in parallel, resulting in lower *g*_s_ of plants grown in elevated CO_2_ when plants from both treatments were measured at the same CO_2_ concentration (Medlyn *et al*., 1999, 2001). Similar results were observed in an experiment with *A. thaliana* in the reproductive stage (Teng *et al.*, 2006). Experiments on earlier growth stages of *A. thaliana*, on the other hand, showed no downregulation of photosynthetic capacity or Rubisco content as long as plants were grown with an ample nitrogen supply (Tocquin *et al.*, 2006; Jauregui *et al.*, 2015). Tocquin *et al.* (2006) suggested that *A. thaliana* under controlled growth conditions simply responds to elevated CO_2_ by growth rate adjustment. One potential explanation for the upregulation of *g*_s_ in our experiment is that increased photosynthetic efficiency under elevated CO_2_ stimulates leaf production and expansion in young *A. thaliana* plants, which not only maintains sink capacity but also increases the need for photosynthate. Consequently, photosynthesis may be stimulated further and result in the observed upregulation of *g*_s_, since changes in leaf CO_2_ demand and supply are typically well coupled (Wong *et al.*, 1979). Indeed, plants grown under elevated CO_2_ in our experiment showed a 60 % increase in total leaf area (data not shown).

Stomatal regulation is a complex, tightly regulated trait of crucial importance for plant fitness and survival. As such, it can be expected to be controlled by the coordinated action of many genes with a certain degree of redundancy. Indeed, we identified numerous loci associated with stomatal regulation in addition to the major QTL on chromosome 2. Most of these QTLs explained minor proportions of the trait variation, but could potentially provide useful information about candidate genes if mapped more precisely. As the main focus of the present study was on the stomatal CO_2_ response, for which phenotyping is very time-consuming, it was necessary to work with a reduced set of lines. However, traits such as water-use efficiency and absolute *g*_s_ could be quantified in a larger population, which may increase mapping power and resolution (Keurentjes *et al.*, 2007). For several traits we identified QTLs with allelic effects opposite to those predicted by the parental phenotypes, corroborating the observation of transgressive segregation in the RIL population.

This study clearly demonstrates the large potential of using natural variation in *A. thaliana* to uncover the genetic basis of stomatal regulation and water economy in plants. The detection of two different QTLs in the same region using mapping populations with separate genetic backgrounds (the present study; Juenger *et al.*, 2005; Brosché *et al.*, 2010; Des Marais *et al.*, 2014; Jakobson *et al.*, 2016) highlights the importance of exploiting the variation among numerous accessions to fully resolve the genetic regulation of complex traits. The large differences in *g*_s_ or water-use efficiency observed among cultivars of wheat (Lu *et al.*, 1998; Condon *et al.*, 2004), rice (Horie *et al.*, 2006), maize (Ryan *et al.*, 2016), legumes (Ehleringer *et al.*, 1991; Ashok *et al.*, 1999) cotton (Lu *et al.*, 1998) and sugarcane (Basnayake *et al.*, 2015) show that there is a large untapped potential in the genetic variation for stomatal traits in crop species as well. Breeding for low *g*_s_ and high water-use efficiency may result in crop varieties suitable for cultivation in already dry areas (Araus *et al.*, 2002), as shown by the successful development of transpiration-efficient wheat cultivars (Rebetzke *et al.*, 2002; Condon *et al.*, 2004). In the more moist and fertile areas currently suitable for highly productive crops with relatively high *g*_s_, it would be advantageous to grow cultivars exhibiting a gradual but substantial shift towards lower *g*_s_ as the atmospheric CO_2_ concentration increases and the air, and perhaps also the soil, becomes progressively drier. While there is typically a trade-off between high gas exchange and high water-use efficiency, our results show that plants with high *g*_s_ at the current atmospheric CO_2_ concentration may also exhibit large improvements in water economy under rising atmospheric CO_2_. In fact, high *g*_s_ at present-day atmospheric CO_2_ concentration and strong stomatal responsiveness to CO_2_ were associated with the same QTL in the present study, and may even be regulated by the same gene.

## SUPPLEMENTARY DATA

Supplementary data are available online at https://academic.oup.com/aob and consist of the following. Figure S1: histogram plots showing the distribution of trait data among RILs. Table S1: genotype data and genetic maps of RILs and NILs. Table S2: sequences and annealing temperatures of primers. Table S3: trait data from RILs grown in ambient CO_2_. Table S4: trait data from RILs grown in ambient and elevated CO_2_ concentration. Table S5: results of fine mapping of a major QTL at the end of chromosome 2.

mcaa065_suppl_Supplement_Table_S1

mcaa065_suppl_Supplement_Table_S2

mcaa065_suppl_Supplement_Table_S3

mcaa065_suppl_Supplement_Table_S4

mcaa065_suppl_Supplement_Table_S5

mcaa065_suppl_Supplement_Figure
