# Supplementary figures and images for: Genetic controls of short- and long-term stomatal CO2 responses in Arabidopsis thaliana
Source: Ann Bot. 2020 Apr 16;126(1):179–90. doi: 10.1093/aob/mcaa065 (PMC7304471; doi:10.1093/aob/mcaa065)

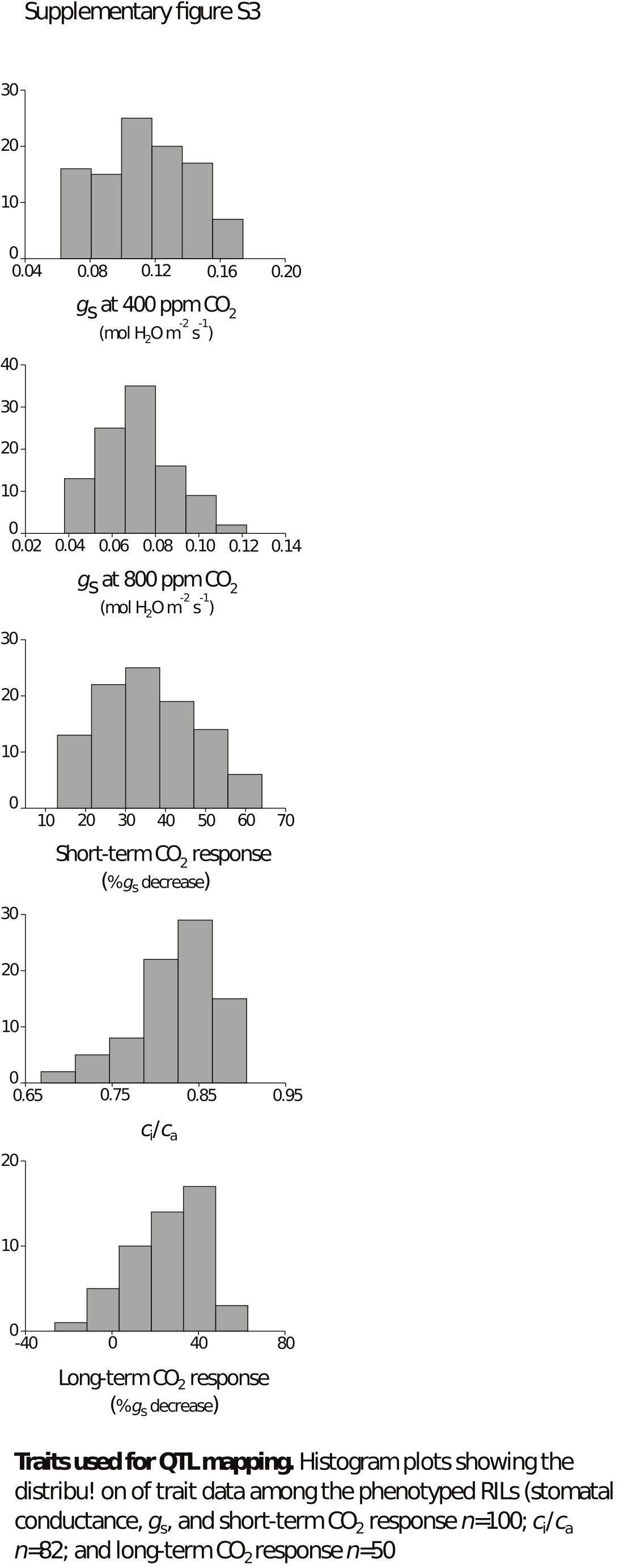

Supplement: mcaa065_suppl_Supplement_Figure [file mcaa065_suppl_supplement_figure.png]
